# Role of diagnostic laparoscopy in patients with large cell neuroendocrine carcinoma of the ovary with cancerous peritonitis: case report and review of the literature

**DOI:** 10.1186/s13048-019-0571-8

**Published:** 2019-10-15

**Authors:** Hideaki Tsuyoshi, Kenji Yashiro, Shizuka Yamada, Makoto Yamamoto, Toshimichi Onuma, Tetsuji Kurokawa, Yoshio Yoshida

**Affiliations:** Department of Obstetrics and Gynecology, Faculty of Medical Sciences, University of Fukui, 23-3 Shimoaizuki, Matsuoka, Eiheiji-cho, Yoshida-gun, Fukui, 910-1193 Japan

**Keywords:** Large cell neuroendocrine carcinoma, Carcinomatous peritonitis, Ascitic fluid cytology, Diagnostic laparoscopy, Decision-making

## Abstract

**Background:**

Large cell neuroendocrine carcinoma is a very rare ovarian neoplasm that has a poor clinical outcome even in the early stage, and there is as yet no established treatment. Diagnostic laparoscopy has been used to determine the possibility of primary optimal cytoreductive surgery or neoadjuvant chemotherapy in patients with advanced epithelial ovarian cancer. However, the role of diagnostic laparoscopy is still unclear in large cell neuroendocrine carcinoma due to its rarity.

**Case presentation:**

A 31-year-old woman with abdominal distention was referred to our hospital. She was strongly suspected of having advanced ovarian cancer because of a huge pelvic mass, massive ascites, and their appearance on medical imaging. However, cytological examinations from ascitic fluid by abdominal paracentesis did not show any malignant cells. She underwent diagnostic laparoscopy to evaluate the possibility of primary optimal cytoreductive surgery, and only tissue sampling was performed for pathological diagnosis because of the countless disseminated lesions of various sizes in the intraperitoneal organs. The patient had no postoperative complications, leading to the early start of postoperative chemotherapy.

**Conclusions:**

To date, there have been no systematic reviews that focused on determining the treatment strategy using laparoscopy. Diagnostic laparoscopy can be helpful to determine the optimal treatment, including primary debulking surgery, neoadjuvant chemotherapy, or best supportive care, assisting in decision-making particularly for patients with advanced large cell neuroendocrine carcinoma with carcinomatous peritonitis.

## Introduction

Neuroendocrine carcinomas, particularly large-cell neuroendocrine carcinoma (LCNEC) of the ovary, are extremely rare but aggressive neoplasms. The other common subtypes of ovarian cancer such as high-grade serous, mucinous, or endometrioid carcinoma are often associated with LCNEC. LCNEC can follow a different clinical course with more adverse outcomes. Early and accurate diagnosis of LCNEC can be the best way to proceed to the optimal treatment and improve the prognosis of these patients. However, there are no optimal diagnostic and treatment strategies because of the paucity of evidence about the clinical or imaging features.

Currently, diagnostic laparoscopy has been used to determine the treatment strategy, including primary optimal cytoreductive surgery or neoadjuvant chemotherapy, in patients with advanced epithelial ovarian cancer, although the role of diagnostic laparoscopy is still unclear in LCNEC. A case of diagnostic laparoscopy with no perioperative complications resulting in the early start of postoperative chemotherapy is presented, along with a review of the literature to explain the role of diagnostic laparoscopy in selecting the optimal treatment for LCNEC patients.

## Case presentation

A 31-year-old woman (gravida 0, para 0) visited the hospital due to abdominal distension. She had been in good health. Physical examination showed a markedly distended abdomen. Abdominal ultrasound (US) showed a pelvic mass and gross ascites. She was referred to our hospital for further examination and subsequent treatment.

Transvaginal US showed the presence of marked ascites and a large solid and cystic mass with a diameter of 20 cm around the uterus in the pelvic cavity. CT of the abdomen and pelvis showed gross ascites that extended under the diaphragm, a strongly enhanced, heterogeneous, huge mass in the pelvic cavity, and multiple peritoneal nodule lesions. There was no lymphadenopathy. On pelvic MRI, the pelvic mass showed homogeneous low intensity on T1-weighted MRI and heterogeneous low and high intensities on T2-weighted MRI, suggesting the presence of cystic and solid lesions. There were no fatty components. The patient’s serum CA 125 level was 165.7 U/ml (normal value < 35 U/ml). Serum CEA and CA 19–9 levels were within normal ranges. The pelvic cyst with solid components, the high CA 125 level, massive ascites, and multiple peritoneal nodule lesions strongly indicated the presence of an advanced ovarian cancer.

**Fig. 1:**
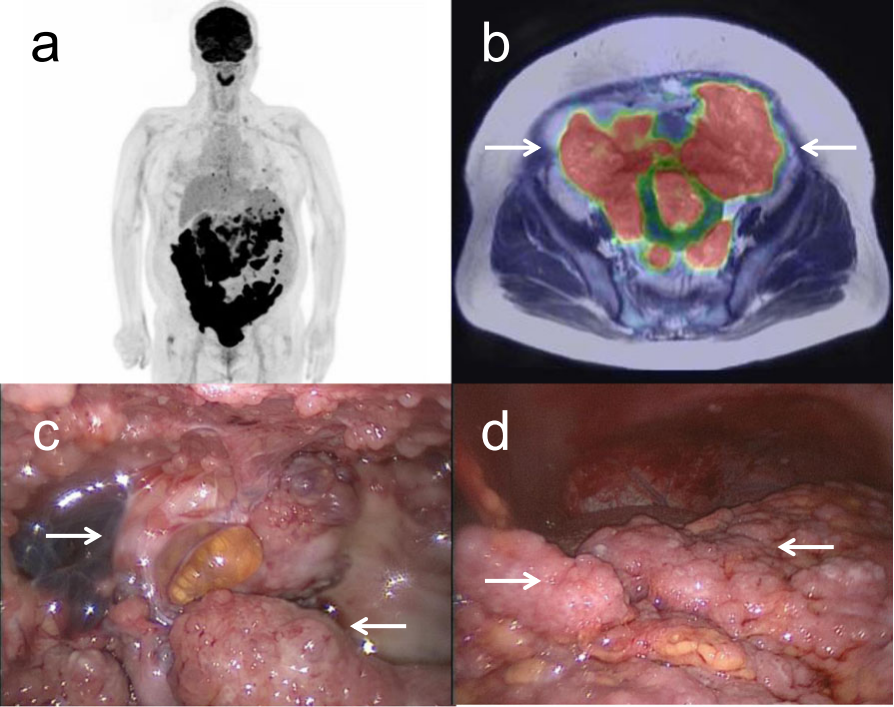
Whole-body FDG PET shows strong FDG uptake in multiple peritoneal nodule lesions (**a**). Integrated FDG-PET/T2-weighted MRI shows strong FDG uptake in the huge mass in the pelvic cavity (arrows) (**b**). Laparoscopic findings show the huge mass with a diameter greater than 20 cm in the pelvic cavity and strongly adhered to the adjacent organs (arrows) (**c**) and the countless disseminated lesions of various sizes in the intraperitoneal organs (arrows) (**d**)

The patient underwent whole-body FDG PET/MRI to confirm malignancy and the presence of lymph node or distant metastases. The huge mass in the pelvic cavity and multiple peritoneal nodule lesions showed strong FDG uptake (Fig. 1a and b). She underwent abdominal paracentesis several times to confirm malignant cells in the ascitic fluid and reduce the abdominal discomfort caused by the massive ascites. However, cytological examinations showed only mesothelial cells without any malignant cells despite the imaging appearance of suspected malignancy on CT, MRI, and FDG-PET.

**Fig. 2:**
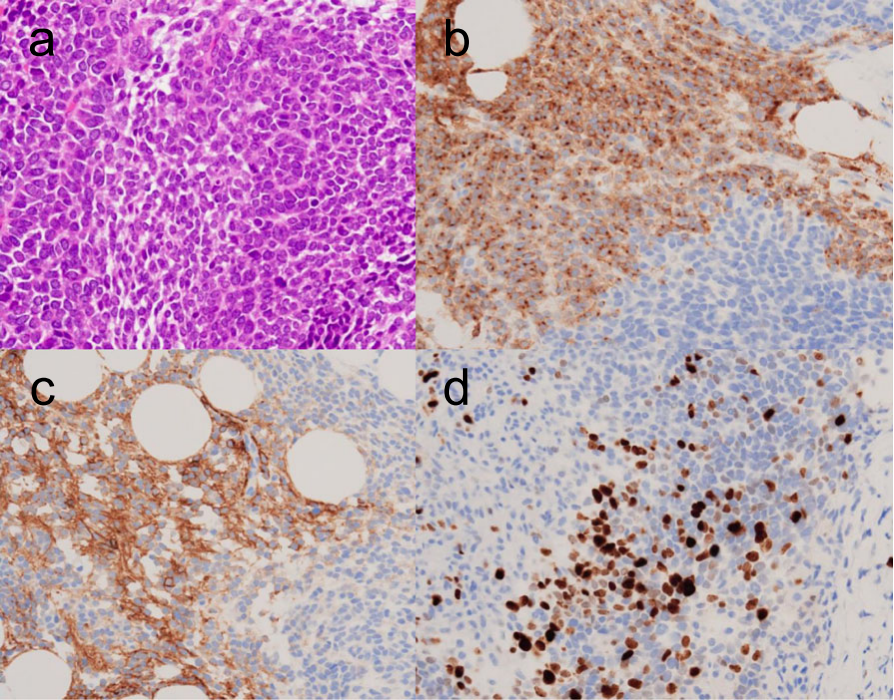
Hematoxylin and eosin-stained paraffin section of the tumor at × 40 magnification shows that the tumor consists of small cells and large cells with hyperchromatic nuclei and a high mitotic rate, with nested, trabecular, and pseudoglandular growth patterns (**a**). Immunohistochemical analysis at × 40 magnification shows that these cells are positive for the neuroendocrine markers, synaptophysin (**b**) and CD56 (**c**). The Ki-67 index is greater than 20% (**d**)

Therefore, exploratory laparoscopy was performed for diagnostic purposes, and 6900 ml of bloody serous ascites were evacuated and obtained for cytology. A huge mass with a diameter of more than 20 cm occupied the pelvic cavity and adhered strongly to adjacent organs including the uterus, adnexa, and rectum (Fig. 1c). It was decided that the optimal surgery could not be performed because there were countless disseminated lesions of various sizes in the intraperitoneal organs, including the omentum, mesentery, and peritoneum (Fig. 1d). Therefore, one nodule of the omentum was resected by a harmonic device for frozen section examination, and it was diagnosed as adenocarcinoma. Additional resection of the omental nodule was performed for permanent fixation and further pathological examination. The pathological examination showed that the tumor consisted of small cells and large cells with hyperchromatic nuclei and a high mitotic rate, showing nested, trabecular, and pseudoglandular growth patterns (Fig. 2a). There was no other histologic subtype. Immunohistochemical analysis showed that these cells were positive for neuroendocrine markers such as synaptophysin and CD56, and the Ki-67 index was greater than 20% (Fig. 2b, c and d). Cytological examination of the obtained ascitic fluid showed no malignant cells. There were no other suspicious malignant lesions in the lung or digestive system on CT or FDG-PET. Therefore, the final pathological diagnosis was large cell neuroendocrine carcinoma of the ovaries with FIGO stage IIIC.

The patient’s postoperative course was uneventful. On the 11th day after surgery, she received the first course of chemotherapy with etoposide and cisplatin. After 2 courses of the chemotherapy, the symptoms of abdominal distension had improved, although there was residual tumor in the abdominal cavity. However, the residual tumor then increased rapidly, and the patient died 2 months after surgery.

## Discussion and conclusions

Neuroendocrine carcinomas (NECs) are rare but aggressive types of neoplasms that are generally seen in the lungs or gastrointestinal tract, whereas they are very rarely seen at sites in the female genital tract including the ovaries. Family history of cancer, body mass index, diabetes mellitus, cigarette smoking and alcohol consumption have been reported as the potential risk factors for NECs of the pancreas, small intestine, and rectum, whereas the risk factors for NECs in the ovaries remain unknown [1]. The 2014 WHO classification of ovarian tumors included carcinoid and small-cell carcinoma, pulmonary type, but no separate category of NEC. Moreover, large-cell NEC arises very rarely in the ovary, although the WHO classification does not include it [2]. NECs account for only 0.1% of all ovarian cancers and are often associated with other epithelial carcinomas, such as high-grade serous, mucinous, or endometrioid carcinoma. Unlike carcinoid, small-cell and large-cell NECs of the ovaries often occur in young women, with a median age of 23.9 years, and are associated with a much poorer prognosis [3]. Surgery with the aim of diagnosis and complete resection and adjuvant chemotherapy with platinum-based chemotherapy have been used regardless of the paucity of data. Pathological examination shows large cells with significant pleomorphism, large nuclei with coarse and granular chromatin, prominent nucleoli, significant mitotic activity, and palisading with rosette formation. Immunohistochemical analysis shows positivity for one or more of the neuroendocrine markers such as synaptophysin, CD56, or chromogranin in at least 10% of tumor cells. In many cases, the early and accurate diagnosis of NEC can be the best way to improve the prognosis of NEC of the ovaries.

**Fig. 3:**
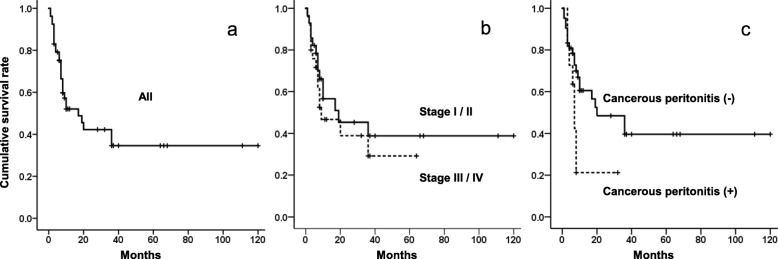
Kaplan-Meier survival curves for overall survival of LCNEC patients. The total 5-year survival is 34.6%, and median survival time is 17 months in all LCNEC patients (*n* = 53) (**a**). There are no significant differences between the cases with FIGO stage I/II (*n* = 28) and III/IV (*n* = 25), with total 5-year survival of 38.8 and 29.2% and median survival time of 19 and 9 months, respectively (*p* = 0.458) (**b**). The cases with carcinomatous peritonitis (*n* = 11) show significantly much worse clinical outcomes than cases without carcinomatous peritonitis (*n* = 42), with median survival time of 20 and 7 months, respectively (*p* = 0.036) (**c**)

The clinicopathological features of 31 cases with FIGO stage I/II (Table 1) [4–24] and 25 cases with FIGO stage III/IV (Table 2) [7, 8, 14, 17, 18, 20, 25–35] were reviewed. A Kaplan-Meier survival curve (SPSS Statistics version 24; IBM, Armonk, NY) of all 53 cases excluding 3 cases without clinical outcome data showed total 5-year survival of 34.6% and median survival time of 17 months. There were no significant differences between the cases with FIGO stage I/II (*n* = 28) and III/IV (*n* = 25), with total 5-year survival of 38.8 and 29.2% and median survival time of 19 and 9 months, respectively (*p* = 0.458). These results were almost similar to the previous report by Oshita et al. [18], suggesting that cases with LCNEC showed much worse clinical outcomes than the other subtypes, such as epithelial ovarian cancer even in early disease, regardless of FIGO stage. In particular, cases with carcinomatous peritonitis (*n* = 11) showed significantly much poorer clinical outcomes than cases without carcinomatous peritonitis (*n* = 42), with median survival time of 7 and 20 months, respectively (*p* = 0.036), suggesting that a different therapeutic strategy should be considered in these cases (Fig. 3).

**Table 1:** Literature review of LCNEC of FIGO stage I and II or undecided

Study	Age (y)	FIGO stage	Size (cm)	Associated component	Metastatic site	Ascitic fluid cytology	Treatment	Outcome
Collins et al., 1991 [4]	34	IC	16	Mucinous cystadenoma & mucinous adenoCa	None	N/A (bloody 4 l)	TAH/BSO/OM + CDDP/CPA	DOD 8 months
Khurana et al., 1994 [5]	22	I	21	Mucinous cystadenoma & mucinous adenoCa	None	N/A	RSO/AP + CBDCA + CPA	DOD 3 months
Jones et al., 1996 [6]	65	IA	16.5	Mucinous cystadenoma	None	Negative (1.6 l)	TAH/BSO/OM/PLN/AP	DOD 10 months
Eichhorn et al., 1996 [7]	77	IA	15	Endometrioid adenoCa grade 1	None	N/A	TAH/BSO/LN & peritoneal biopsies	DOD 19 months
	36	IA	10	Mucinous adenoCa	None	N/A	RSO/AP	Recent
	45	IB	18	Mucinous borderline tumor with small foci of mucinous adenoCa	None	N/A	TAH/BSO/OM + Chem	DOD 36 months
	68	IIB	9	Mucinous adenoCa	Right tube	N/A	TAH/RSO/OM/peritoneal biopsy	Lost to follow-up
Chen, 2000 [8]	44	IA	25	Mucinous intraepithelial adenoCa	None	N/A	TAH/BSO/OM + PTX + CBDCA	DOD 4 months
Behnam et al., 2004 [9]	27	IA	17	Pure LCNEC	None	N/A	LSO/right OV & pelvic wall biopsies/PAN//OM/AP + PTX + CBDCA	NED 10 months
Hirasawa, 2004 [10]	56	IIC	18	Mucinous adenoCa & dermoid cyst	Rectum	Positive (adenoCa)	TAH/BSO/rectal serosa resection/PLN	DOD 10 months
	35	IC	N/A	Mucinous adenoma	None	N/A	TAH/BSO/OM + CDDP (ip) + high-dose Chem	NED 10 years
Ohira et al., 2004 [11]	33	IC	11	Endometrioid adenoCa grade 1	None	Ruptured	LSO/right OV biopsy/OM + CPT-11/Nedaplatin	DOD 6 months
Ahmed et al., 2005 [12]	31	N/A	15	Mucinous cystadenoma	N/A	N/A	N/A	N/A
Lindboe, 2007 [13]	64	IA	14	Pure LCNEC	None	Negative	TAH/BSO/OM + Bleomycin/CDDP/Etoposide	NED 9 months
Veras et al., 2007 [14]	55	I	26	Mucinous low malignant potential with intraepithelial Ca	N/A	N/A	TAH/BSO + platinum-based Chem	NED 68 months
	54	I	14	Mucinous & endometrioid Ca	N/A	N/A	TAH/BSO + platinum-based Chem	NED 66 months
	59	I	14	High-grade adenoCa, not otherwise specified	N/A	N/A	BSO + platinum-based Chem	NED 28 months
	22	I	21	Mucinous low malignant potential with mucinous Ca	N/A	N/A	RSO/AP + platinum-based Chem	DOD 3 months
Tartaglia et al., 2008 [15]	56	IIA	8	Pure LCNEC	Endometrium	Negative	TAH/BSO/pelvic wall biopsies/OM/AP/PAN + PTX/CBDCA	NED 10 months
Aslam et al., 2009 [16]	76	IIB	30	Pure LCNEC	Douglas pouch	N/A	TAH/BSO/OM/AP/PLN/PAN/Douglas pouch resection	DOD post operation
Chenevert et al., 2009 [17]	53	I	21	Mucinous adenoCa & dermoid cyst	None	N/A	TAH/BSO/OM/PLN + CDDP/etoposide	DOD 7 months
Oshita et al., 2011 [18]	80	IIC	7	Endometrioid adenoCa	Left tube, parametrium	Ruptured	TAH/BSO/PLN/OM /AP + PTX + CBDCA	NED 40 months
	65	IC	11	Endometrioid adenoCa with squamous differentiation	None	Ruptured	TAH/BSO/OM + PTX/CBDCA	DOD 2 months
Lee et al., 2012 [19]	40	IA	30	Mucinous Ca	None	N/A	TAH/BSO/RPLN/OM/AP + PTX/CBDCA	NED 8 months
Ki et al., 2014 [20]	58	IA	N/A	Pure LCNEC	None	N/A	TAH/BSO/OM/PLN + PTX/CDDP	DOD 17 months
	67	IIB	13	Pure LCNEC	Pelvic peritoneum	N/A	TAH/BSO/RPLN/OM + PTX/CBDCA	NED 5 months
Asada et al., 2014 [21]	50	IA	15	Mucinous adenoma	None	N/A	TAH/BSO/PLN/OM + etoposide/CDDP	DOD 7 months
Ding et al., 2014 [22]	70	IA	16	Borderline mucinous tumor	None	N/A	TAH/BSO/PLN/OM/AP	NED 6 months
Sehouli et al., 2016 [23]	23	II	N/A	N/A	N/A	N/A	TAH/BSO/RPLN/OM/AP/colon resection + PTX/CBDCA	NED 111 months
	61	I	N/A	N/A	N/A	N/A	TAH/BSO	NED 37 months
Doganay et al., 2019 [24]	73	II	10	Pure LCNEC	Uterus, bladder, rectum	Positive (malignant cells)	TAH/BSO/pelvic mass resection + etoposide/CDDP	AEW 6 months

**Table 2:** Literature review of LCNEC of FIGO stage III and IV

Study	Age (y)	FIGO stage	Size (cm)	Associated component	Metastatic site	Ascitic fluid cytology	Treatment	Outcome
Eichhorn et al., 1996 [7]	58	IIIB	30	Mucinous borderline tumor with small foci of mucinous adenoCa	Appendix, peritoneum	N/A	TAH/BSO/OM/AP/LN & peritoneal biopsies + Chem	DOD 8 months
Chen, 2000 [8]	73	IIIC	11	Microinvasive mucinous adenoCa	Small bowel serosa, retroperitoneal LN	N/A	BSO/OM/RPLN+ PTX/CDDP	DOD 8 months
Choi et al., 2007 [25]	71	IIIB	6.5	Serous Ca	Retroperitoneum	N/A	TAH/BSO + PTX/CBDCA	NED 8 months
Veras et al., 2007 [14]	39	IV	26	Mucinous adenoCa	N/A	N/A	TAH/BSO + platinum-based Chem	AWD 8 months
	42	IV	N/A	Benign cyst & dermoid cyst in contralateral OV	N/A	N/A	TAH/BSO + platinum-based Chem	DOD 20 months
	53	III	14.5	Endometrioid adenoCa	N/A	N/A	TAH/BSO + platinum-based Chem	NED 37 months
	47	III	14	AdenoCa, not otherwise specified & mature teratoma	N/A	N/A	TAH/BSO + platinum-based Chem	NED 11 months
	25	IV	5	Mature cystic teratoma	N/A	N/A	BSO/OM/AP + platinum-based Chem	DOD 36 months
	55	III	13.5	Mucinous low malignant potential	N/A	N/A	TAH/BSO + platinum-based Chem	DOD 2 months
	63	IV	14	Endometrioid adenoCa	N/A	N/A	TAH/RSO + platinum-based Chem	DOD 9 months
Tsuji et al., 2008 [26]	46	IIIC	12	Squamous differentiation	Omentum, peritoneum, rectum, uterus	N/A (bloody 2 l)	subTAH/BSO/OM + PTX/CBDCA	DOD 4 months
Dundr et al., 2008 [27]	73	IV	9	Pure LCNEC	Mesentery, left renal capsule, CNS	N/A	CNS meta resection/γ Knife + TAH/BSO/OM/mesenterial meta resection/left nephrectomy + PTX/CBDCA + γ Knife	NED 12 months
Chenevert et al., 2009 [17]	53	20	IV	Mucinous adenoCa	Douglas pouch, lungs, mediastinal LN, liver, bone	N/A	TAH/BSO/OM/PLN/Douglas pouch resection + PTX/CBDCA	DOD 3 months
Yasuoka et al., 2009 [28]	36	26	IIIA1	Mucinous intraepithelial Ca	Periaortic LN	N/A	TAH/BSO/RPLN/OM + Chem	NED 6 months
Draganova-Tacheva et al., 2009 [29]	68	IV	18	Serous Ca	Peritoneum, omentum, uterus, bladder, colon, diaphragm, inguinal LNs	Positive (papillary serous adenoCa)	NAC (PTX/CBDCA) + IDS (BSO/OM) + PTX/CBDCA	DOD 7 months
Oshita et al., 2011 [18]	66	IV	11	N/A	Vagina, lungs	N/A	NAC (PTX/CBDCA) + IDS (TAH/BSO/OM/peritoneal biopsy) + PTX/CBDCA + brain meta resection & RT	NED 64 months
	42	IIIB	13	Endometrioid adenoCa	Peritoneum	N/A	TAH/BSO/RPLN/OM/Douglas pouch resection+ PTX/CBDCA	NED 32 months
Miyamoto et al., 2012 [30]	69	IV	15	Mature cystic teratoma	Peritoneum, lungs, retroperitoneal & subclavian LNs	Ruptured	LSO/RPLN/subclavian LN biopsies + PTX/CBDCA	DOD 6 months
Shakuntala et al., 2012 [31]	40	IIIC	20	Pure LCNEC	Omentum, paraaortic LN, bowel, bladder	N/A (minimal bloody)	BSO/OM/PAN/bladder & sigmoid colon deposit resection + CDDP/etoposide	NED 6 months
Ki et al., 2014 [20]	77	IV	15	Pure LCNEC	Supraclavicular LN	Positive (malignant cells)	TAH/pelvic & neck masses resection + etoposide/CBDCA	DOD 45 days
Cokmert et al., 2014 [32]	68	IV	20	AdenoCa	Uterus, bladder, sigmoid colon, omentum, lungs mediastinum LN, bones	N/A	TAH/BSO/RPLN/OM + RT	DOD 7 months
Lin et al., 2014 [33]	50	IV	25	Pure LCNEC	Liver, pelvic wall, intestine, left tube, parametrium, omentum, appendix	N/A	TAH/BSO/OM + PTX/CBDCA	DOD 3 months
Agarwal et al., 2016 [34]	35	IIIC	6	Pure LCNEC	Cervix, retroperitoneal LN	Negative	TAH + BSO	AWD 3 months
Herold et al., 2018 [35]	75	IV	13	Pure LCNEC	Pelvic peritoneum, retroperitoneal LN, liver	N/A	Laparoscopy → BSO/RPLN/OM/pelvic peritoneum & liver resection + etoposide/CBDCA + PTX/CBDCA	NED 36 months
Present	31	IIIC	10	Pure LCNEC	Peritoneum, omentum	Negative (bloody 7 l)	Diagnostic laparoscopy + etoposide/CDDP	DOD 3 months

*LCNEC* large cell neuroendocrine carcinoma, *Ca* carcinoma, *FIGO* International Federation of Gynecology and Obstetrics, *TAH* total abdominal hysterectomy, *BSO* bilateral salpingo-oophorectomy, *RSO* right salpingo-oophorectomy, *LSO* left salpingo-oophorectomy, *RPLN* retroperitoneal lymphadenectomy, *PLN* pelvic lymphadenectomy, *PAN* para-aortic lymphadenectomy, *OM* omentectomy, *AP* appendectomy, *IDS* interval debulking surgery, *OV* ovary, *LN* lymph node, *CNS* central nervous system, *meta* metastasis, *RT* radiation, *Chem* chemotherapy, *CDDP* cisplatin, *CPA* cyclophosphamide, *CBDCA* carboplatin, *PTX* paclitaxel, *ADR* adriamycin, *CPT-11* irinotecan, *NAC* neoadjuvant chemotherapy, *ip* intraperitoneal, *DOD* dead of disease, *NED*: no evidence of disease, *AWD* alive with disease, *N/A* no information available

In advanced epithelial ovarian cancer patients with cancerous peritonitis and ascites, ascitic fluid cytology by abdominal paracentesis before surgery can be helpful and less invasive to confirm malignant cells in the ascitic fluid to proceed to neoadjuvant chemotherapy followed by interval debulking surgery with improvement of the prognosis [36]. In the present case, the patient was strongly suspected of having advanced ovarian cancer because of the huge pelvic mass, massive ascites, and their appearance on medical imaging. However, cytological examinations from ascitic fluid by abdominal paracentesis did not show any malignant cells. The previous study had shown that 96.7% of patients with carcinomatous peritonitis had positive ascitic fluid cytology [37]. In our review, only 2 of 5 cases with stage I/II and 2 of 4 cases with stage III/IV had positive ascitic fluid cytology. Only in 2 cases with carcinomatous peritonitis, including the present case, ascitic fluid cytology has been reported. The present case did not show any malignant cells in the ascitic fluid, whereas another one showed malignant cells consistent with papillary serous adenocarcinoma, which was the associated component in this case [29]. A previous study showed that the adenocarcinoma components were predominately located at the surface of the tumors, and most stromal and vascular invasion and lymph node metastases involved neuroendocrine components in the mixed adenoneuroendocrine carcinomas of hepatobiliary organs [38], suggesting that it might be difficult to identify malignant cells derived from pure LCNEC in ascitic fluid. Therefore, pathological examination has to be performed to differentiate non-epithelial ovarian cancer or other diseases including inflammation in these LCNEC cases. In the previous cases with stage III/IV [7, 8, 14, 17, 18, 20, 25–34], most patients underwent laparotomy for diagnosis and treatment. However, complete surgery could not be performed in many cases, leading to the deterioration of the patients’ general condition and much poorer outcomes. Therefore, a non-invasive method for differential diagnosis is needed.

The role of diagnostic laparoscopy to determine the possibility of primary optimal cytoreductive surgery in patients with advanced epithelial ovarian cancer has been reported. In a randomized, controlled trial involving patients with suspected advanced ovarian cancer, diagnostic laparoscopy was reported to reduce the number of futile laparotomies and be reasonable to proceed with primary cytoreductive surgery if cytoreduction to less than 1 cm of residual disease seems feasible [39]. Moreover, the same group has reported that diagnostic laparoscopy did not increase total direct medical health care costs or adversely affect complications or quality of life [40], suggesting that laparoscopy might be a potential diagnostic procedure in advanced epithelial ovarian cancer, although port-site metastasis occurs in 16–47% of cases, and the prognostic impact is still controversial [41].

In the previous cases with LCNEC, Herold et al. reported that diagnostic laparoscopy can be useful to achieve complete primary debulking surgery leading to better outcomes [35]. Oshita et al. reported the usefulness of neoadjuvant chemotherapy, leading to complete interval debulking surgery and long survival [18]. In the present case, the patient underwent diagnostic laparoscopy, and it was decided that the optimal surgery could not be performed because of the countless peritoneal lesions. Therefore, tissue sampling was performed without any intraoperative complications. The patient also had no postoperative complications, leading to the early start of postoperative chemotherapy. In terms of the chemotherapeutic regimen, the regimen against the epithelial component including paclitaxel and carboplatin could be considered in cases of mixed epithelial and LCNEC ovarian tumors, whereas the regimen against the neuroendocrine component including platinum-etoposide could be considered in cases of pure LCNEC [3, 42]. Therefore, the patient received the chemotherapy with etoposide and cisplatin, although postoperative chemotherapy did not improve the clinical outcome in the present case with carcinomatous peritonitis. Previous reports have shown the possible efficacy of paclitaxel and carboplatin, which can be less toxic than cisplatin in cases of even pure LCNEC of the ovary [9, 15, 20, 27, 33, 35], as well as in cases of NEC of the uterine cervix [43], suggesting that these chemotherapeutic regimens could be considered in patients with poor performance status and prognosis because of unresectable carcinomatous peritonitis. Taken together, in LCNEC, diagnostic laparoscopy followed by primary debulking surgery or neoadjuvant chemotherapy might be useful in cases without carcinomatous peritonitis, whereas it might also be useful for deciding whether patients should receive less invasive chemotherapy or best supportive care in cases with carcinomatous peritonitis with much poorer outcomes.

In summary, diagnostic laparoscopy could facilitate determination of subsequent treatment, including primary debulking surgery or neoadjuvant chemotherapy, in LCNEC patients. Moreover, it can also be useful for deciding whether to give adjuvant treatment or best supportive care to LCNEC patients with carcinomatous peritonitis who show much worse clinical outcomes.

## Data Availability

The data supporting the findings of this study are available within the article.

## Abbreviations

CT: Computed tomography
FDG: Fluorodeoxyglucose
FIGO: International Federation of Gynecology and Obstetrics
LCNEC: Large cell neuroendocrine carcinoma
MRI: Magnetic resonance imaging
PET: Positron emission tomography
US: Ultrasonography
WHO: World Health Organization
